# Diagnostic performance of mono-exponential DWI versus diffusion kurtosis imaging in breast lesions: A meta-analysis

**DOI:** 10.1097/MD.0000000000031574

**Published:** 2022-11-04

**Authors:** Yewu Wang, Yumei Jin, Mou Li, Jun Zhang, Shaoyu Wang, Huapeng Zhang, Bin Song

**Affiliations:** a Department of Joint and Sports Medicine, Qujing First People’s Hospital, Qujing, Yunan Province, China; b Department of Medical Imaging Center, Qujing First People’s Hospital, Qujing, Yunan Province, China; c Department of Radiology, West China Hospital of Sichuan University, Chengdu, Sichuan Province, China; d Department of Radiology, The First Affiliated Hospital of Zhejiang University School of Medicine, Hangzhou, Zhejiang Province, China; e Siemens Medical System Co., LTD, Magnetic Resonance Imaging Research Department, Shanghai, China.

**Keywords:** breast lesions, diagnostic performance, DKI, DWI, meta-analysis

## Abstract

This meta-analysis aimed to explore the diagnostic value of diffusion kurtosis imaging (DKI) compared to mono-exponential diffusion weighted imaging (DWI) in the diagnosis of breast cancer.

**Methods:**

A systematic electronic literature search (up to September 2020) was conducted for published English-language studies comparing the diagnostic values of DKI and DWI for the detection of breast cancer. The data of mean kurtosis (MK), mean diffusivity (MD), and apparent diffusion coefficient (ADC) were extracted to construct 2 *×* 2 contingency tables. The pooled sensitivities, specificities, and areas under the receiver operating characteristic curve (AUCs) were compared between DKI and DWI in the diagnosis of breast cancer.

**Results:**

Eight studies were finally included, with a total of 771 patients in the same population. Pooled sensitivities were 82.0% [95% confidence interval (95% CI), 78.2‐85.3%] for ADC, 87.3% (95% CI, 83.9‐90.1%) for MK, and 83.9% (95% CI, 80.2‐87.1%) for MD. Pooled specificities were 81.1% (95% CI, 76.7‐84.9%) for ADC, 85.1% (95% CI, 81.1‐88.5%) for MK, and 83.2% (95% CI, 79.0‐86.8%) for MD. According to the summary receiver operator characteristic curve analyses, the AUCwas 0.901 for ADC, 0.930 for MK, and 0.918 for MD (ADC vs MK, *P* = .353; ADC vs MD, *P* = .611). No notable publication bias was found, while significant heterogeneity was observed.

**Conclusions:**

Although DKI is feasible for identifying breast cancer, MD and MK offer similar diagnostic performance to ADC values. Thus, we recommend that DKI should not be included in the routine evaluation of breast lesions now.

## 1. Introduction

Breast cancer is the most common cancer in women worldwide.^[1]^ For women with high risk of breast cancer, magnetic resonance imaging (MRI) has been proven to be an important tool for the examination of suspicious breast lesions,^[2]^ as MRI provides multi-directional and multi-sequence imaging along with high soft tissue discrimination ability and can define the nature of the lesions through the blood supply situation around the mass.^[3]^ More importantly, as a functional method of imaging, MRI can reflect the diffusion of water molecules in living tissues by the apparent diffusion coefficient (ADC). Diffusion-weighted imaging (DWI) has been widely used for diagnosis, prognosis, efficacy monitoring and evaluation of recurrence and metastasis.^[4–6]^

However, more and more authors indicted that the movement of water molecules in breast tissue was affected by many factors and did not follow the Gaussian distribution in heterogeneous state, and DWI imaging model cannot full reveal the motion of molecules.^[7,8]^ Jensen et al^[9]^ first introduced a non-Gaussian diffusion model called diffusion kurtosis imaging (DKI) in 2005. DKI was an extension of mono-exponential DWI, which based on the non-Gaussian properties of tissues, came by diffusing kurtosis information to describe the complex microstructure of tissues, theoretically DKI is more consistent with the reality of human tissues than DWI.^[9]^ Several previous studies also^[10–19]^ have evaluated the diagnostic value of DKI compared to DWI for the differentiation of breast lesions, and some of their results were not consistent. Thus, it’s still uncertain whether DKI can outperform mono-exponential DWI in the diagnosis of breast cancer.

Therefore, the purpose of this study was to compare the diagnostic performance of DKI and DWI, so as to show the possibility of DKI as an alternative method of DWI in the diagnosis of breast cancer.

## 2. Materials and Methods

### 2.1. Search strategy

This study did not require ethics approval from our institutional ethics committee, because of the design of this study (meta-analysis).

A systematic literature search on PubMed, Web of Science, and Cochrane Library (Wiley), IEEE, Elsevier, from September 2010 to July 2021, was conducted to identify studies exploring the value of DKI and DWI in the differential diagnosis of breast lesions. The search strategy used the combination of the following medical subject headings terms and keywords: “ADC” AND “DKI OR diffusion kurtosis imaging” AND “breast.” This search was limited to English language publications. The reference lists of finally included articles were assessed for potential inclusion.

### 2.2. Inclusion and exclusion criteria

Studies were eligible for inclusion if both DKI and DWI were performed in the same population for the differentiation of breast lesions, if raw data was sufficient to complete a 2 *×* 2 contingency table, and if the diagnosis was finally confirmed at pathology or follow-up. We excluded these types of studies including animal experiments, letters, reviews, case reports, or proceedings.

### 2.3. Data extraction and quality assessment

Retrieved hits were evaluated for possible inclusion in the study independently by 2 researchers. Any disagreements were resolved through discussion with a third researcher. The included studies were assessed for the required data. First, characteristics were extracted from each study in a standard format: author, year of publication, study design, field strength, estimation method (region of interest or volume of interest), number of malignant and benign lesions, number of b-values, maximum b-values, reference standard, number of patients. Then true-positive (predicted to be malignant, actually malignant), false-positive (predicted to be malignant, actually benign), false-negative (predicted to be benign, actually malignant), and true-negative (predicted to be benign, actually benign) results of ADC, mean kurtosis (MK), and mean diffusivity (MD) were extracted or calculated to build the 2 *×* 2 contingency tables. The quality of the included studies was assessed by the Quality Assessment of diagnostic accuracy studies tool-2^[20]^ using Review Manager Version 5.3.

### 2.4. Statistical analysis

Heterogeneity among included studies was indicated by value calculated by Q statistic of the chi-square test. If there was notable heterogeneity, the pooled sensitivity and specificity were calculated using the random-effect model. The positive likelihood ratio, negative likelihood ratio, and diagnostic odds ratio were also calculated as outlined above. Summary receiver operating characteristic (SROC) curves were built to calculate areas under the receiver operating characteristic curve (AUCs), which were used to evaluate the diagnostic performance of ADC, MK and MD. The *Z* test was performed to compare the difference of statistical significance in AUCs value, and further to assess diagnostic value of DKI and DWI in the diagnosis of breast cancer.

The threshold effect analysis, subgroups analysis and meta-regression analysis were used to explore potential sources of heterogeneity. The threshold effect was assessed by the Spearman correlation coefficient between Logit (sensitivity) and Logit (1-specificity), and *P* value < 0.05 suggested a threshold effect. Publication bias was evaluated by using Deek funnel plot, and *P* value < 0.05 could indicate the existence of publication bias. The above statistical analyses were carried out in Meta-DiSc (version 1.4).

## 3. Results

### 3.1. Literature search

**Figure 1.**
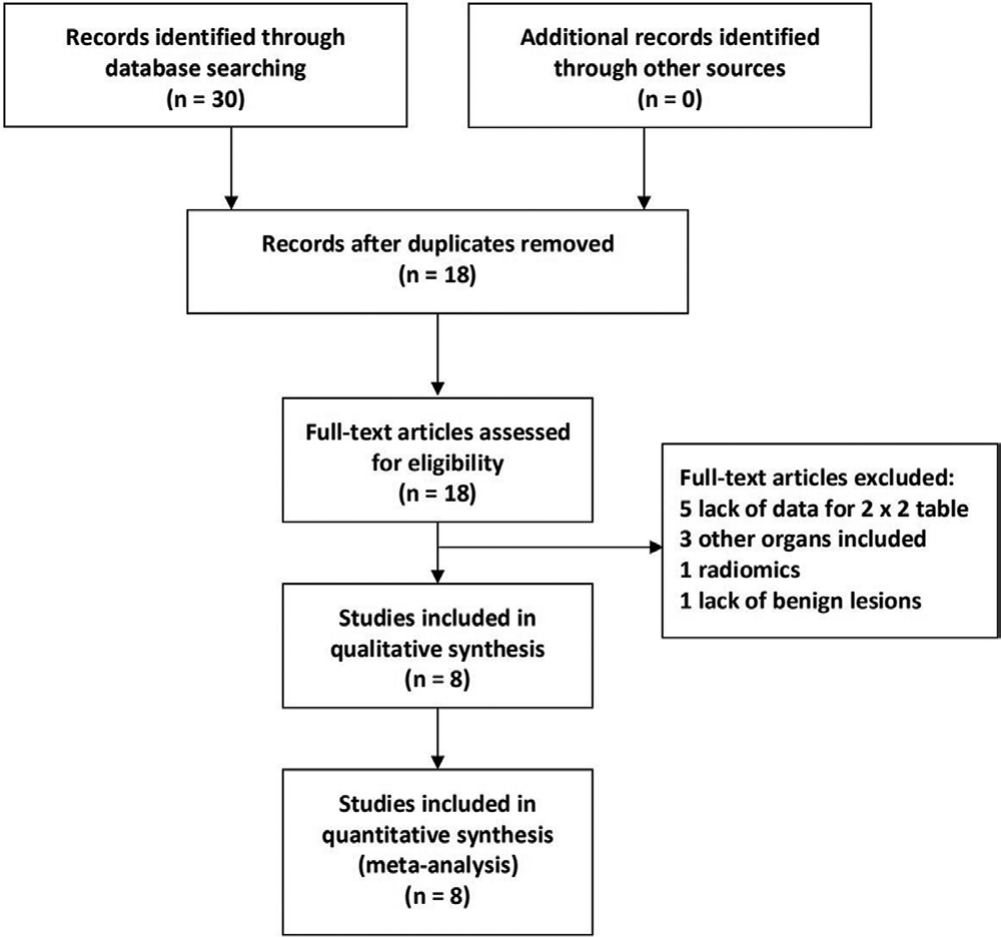
Flowchart of studies’ screening.

**Figure 2.**
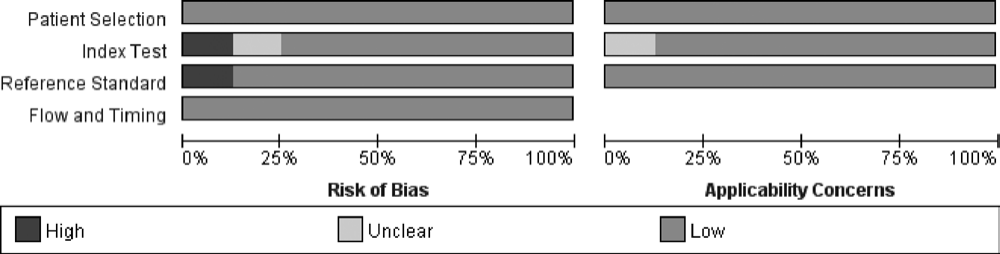
Assessment of the quality of included studies.

We identified 30 abstracts through the initial literature search, and 12 abstracts were removed before the full text assessment. During the full text assessment, 10 studies were eliminated due to lack of sufficient data of DKI and DWI (n = 5), other organs included (n = 3), radiomics based on DKI (n = 1), and lack of benign lesions (n = 1) (Fig. 1). Baseline characteristics of the included studies were summarized in Table 1. The quality of the included studies was good according to the diagnostic accuracy studies tool-2 results (Fig. 2).

**Table 1:** Characteristics of 8 studies included in this meta-analysis for the characterization of breast lesions.

Author	Year	Study design	Field strength	Estimation method	Malignant lesions (n)	Benign lesions (n)	No. of *b* values	Maximum *b* value	*b* values of ADC	Reference standard	No. of patients
Sun et al^[8]^	2015	retro	1.5T	ROI	52 IDC, 5 DCIS	36 FA, 5 others	5	2800	50, 1000	pathology	97
Suo et al^[10]^	2017	retro	3.0T	VOI	44 IDC, 5 ILC, 8 DCIS	27 FA, 4 hyperplasia, 13 others	7	2500	0, 1500	Pathology	101
Li et al^[11]^	2018	retro	3.0T	ROI	62 (no data)	58 (no data)	7	3000	0, 1000	pathology	106
Huang et al^[12]^	2018	pro	3.0T	ROI	44 IDC, 3 ILC, 5 others	19 FA, 7 others	6	2500	0, 800	pathology	71
Liu et al^[13]^	2019	retro	3.0T	VOI	42 (no data)	30 (no data)	4	2000	0, 800	pathology	71
Palm et al^[5]^	2019	retro	3.0T	ROI	44 IDC, 4 DCIS, 17 ILC, 3 others	41 cysts, 19 FA	4	1500	50, 750	Pathology, follow-up	85
Zhou et al^[14]^	2019	pro	1.5T	ROI	59 IDC, 16 DCIS, 13 others	17 FA, 13 inflammation, 14 others	5	2400	50, 1000	pathology	120
Li et al^[15]^	2019	retro	3.0T	VOI	42 IDC, 9 DCIS, 11 others	25 FA, 20 hyperplasia, 13 others	7	3000	0, 1000	pathology	120

ADC = apparent diffusion coefficient, DCIS = ducal carcinomas in situ, FA = fibroadenomas, IDC = infiltrating ductal carcinomas, ILC = infiltrating lobular carcinomas, LCIS = lobular carcinoma in situ, ROI = region of interest, VOI = volume of interest, b-value: the diffusion-weighted signal intensity.

### 3.2. Quantitative synthesis

**Figure 3.**
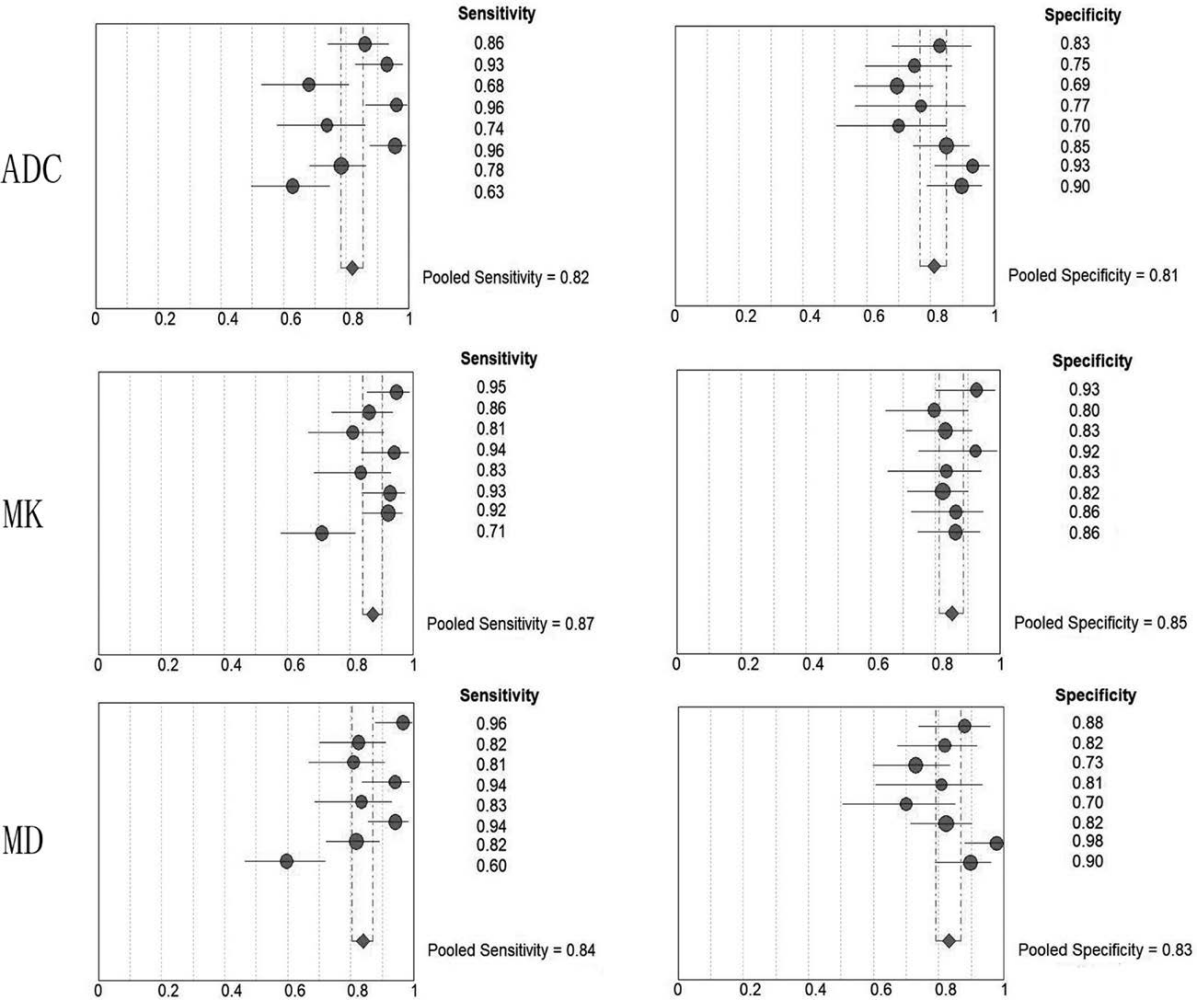
Pooled sensitivity and specificity of ADC, MK, MD. ADC = apparent diffusion coefficient, MD = mean diffusivity, MK = mean kurtosis.

**Figure 4.**
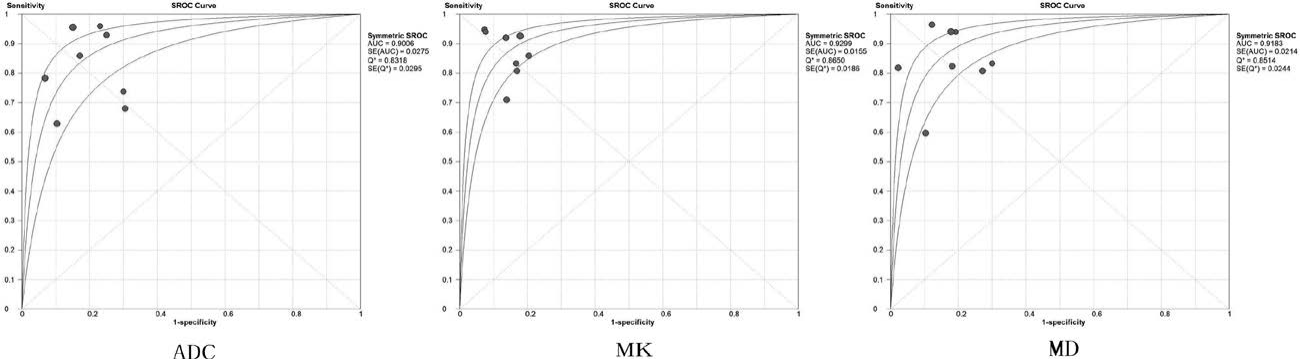
ROC analyses of ADC, MK, MD. ADC = apparent diffusion coefficient, MD = mean diffusivity, MK = mean kurtosis, ROC = receiver operator characteristic curve.

With the absence of threshold effect, we used the random-effect model to pool the sensitivity, and specificity outcomes. Pooled sensitivities were 82.0% (95% CI, 78.2‐85.3%) for ADC, 87.3% (95% CI, 83.9‐90.1%) for MK, and 83.9% (95% CI, 80.2‐87.1%) for MD. Pooled specificities were 81.1% (95% CI, 76.7‐84.9%) for ADC, 85.1% (95% CI, 81.1‐88.5%) for MK, and 83.2% (95% CI, 79.0‐86.8%) for MD (Table 2, Fig. 3). According to the summary receiver operator characteristic curve analyses, the AUC was 0.901 for ADC, 0.930 for MK, and 0.918 for MD (ADC vs MK, *P* = .353; ADC vs MD, *P* = .611). As shown in Table 2 and Figure 4.

**Table 2:** Diagnostic performance of the included 8 studies using both DKI and DWI for the characterization of breast lesions.

	DKI		DWI
Parameter	MK	MD	ADC
No. of TP results	411	395	386
No. of FP results	56	63	71
No. of FN results	60	76	85
No. of TN results	319	312	304
Sensitivity (%) (95% CI)	87.3% (83.9–90.1%)	83.9% (80.2–87.1%)	82.0% (78.2–85.3%)
Specificity (%) (95% CI)	85.1% (81.1–88.5%)	83.2% (79.0–86.8%)	81.1% (76.7–84.9%)
PLR	5.44	4.69	4.20
NLR	0.14	0.17	0.20
DOR	41.1	32.8	24.5
AUC (95% CI)	0.930 (0.900–0.960)	0.918 (0.876–0.960)	0.901 (0.847–0.955)

AUC = area under the summary receiver operating characteristic curve, CI = confidence interval, DOR = diagnostic odds ratio, FN = false-negative, FP = false-positive, NLR = negative likelihood ratio, PLR = positive likelihood ratio, TP = true-positive, TN = true-negative.

### 3.3. Meta-regression and subgroup analysis

The meta-regression analysis indicated that field strength (1.5T vs 3.0T) contributed significantly to the heterogeneity for MD among the included studies (*P* = .04). Then we performed subgroup analyses between different study characteristics such as study design, field strength, maximum b-value, number of b-values, and estimation method. As shown in Table 3, the subgroup analysis showed that studies with fewer b-values (≤5) had higher AUC than that of studies with 6 or 7 b-values (*P* = .047 for MD, no statistical significance for MK). The studies with a maximum b-values of 2000-2500 s/mm^2^ had higher AUC than those with a maximum b-value > 2500 s/mm^2^, with no statistical significance.

**Table 3:** The results of the meta-regression and subgroup analyses in this meta-analysis.

Characteristic	No. of Studies	ADC			*P*	MK			*P*	MD			*P*
		SEN	SPE	AUC		SEN	SPE	AUC		SEN	SPE	AUC	
Study design		.			0.25				0.19				0.19
Pro	2	...	...	...		...	...	...		...	...	...	
Retro	6	0.81	0.80	0.87		0.85	0.84	0.91		0.83	0.81	0.89	
Field strength					0.31				013				***0.04***
1.5 T	2	...	...	...		...	...	...		...	...	...	
3.0 T	6	0.82	0.79	0.87		0.85	0.84	0.91		0.82	0.80	0.88	
Maximum *b* value (s/mm^2^)					0.40				0.49				0.67
1500 ≤ b < 2000	1	...	...	...		...	...	...		...	...	...	
2000 ≤ b ≤ 2500	4	...	...	...		0.90	0.85	0.93		0.85	0.84	0.92	
>2500	3	...	...	...		0.82	0.87	0.92		0.78	0.83	0.90	
Number of *b* values					0.53				0.38				0.23
≤5	4	...	...	...		0.91	0.86	0.94		0.89	0.85	0.95	
>5	4	...	...	...		0.82	0.85	0.91		0.78	0.81	0.88	
Estimation method					0.51				0.22				0.14
VOI	3	0.76	0.80	0.87		0.80	0.83	0.89		0.74	0.83	0.86	
ROI	5	0.85	0.82	0.91		0.91	0.86	0.94		0.89	0.84	0.95	

Of note: *P* values were calculated for the meta-regression analysis.

AUC = areas under the receiver operating characteristic curve, ROI = region of interest, SEN = sensitivity, SPE = specificity, VOI = volume of interest.

### 3.4. Publication bias

Publication bias is assessed visually by using a scatter plot of the inverse of the square root of the effective sample size versus the diagnostic log odds ratio. The Deeks funnel plot regression showed that publication bias was not statistically significant for ADC (*P* = .46), MK (*P* = .59), and MD (*P* = .68).

## 4. Discussion

The findings of our study have shown that DKI was a valuable tool in differentiating malignant from benign breast lesions. Compared with ADC values, the MK and MD values showed higher AUC. However, there were no statistically significant differences, which yielded that neither technique was superior to the other.

Theoretically, DKI, which quantifies non-Gaussian diffusion, is believed to better characterize tissue micro-structure than mono-exponential DWI. DKI model potentially better reflects water diffusion in tissues at ultra-high b-values. Some studies have compared DKI with DWI in the qualitative diagnosis of breast lesions, but the results are inconsistent. Therefore, it is of great significance to summarize the published research results for evaluating the ability of DKI and making a comprehensive comparison with DWI in the diagnosis of breast cancer.

Mammography is the traditional method of breast examination, which is sensitive to calcification. Mammography depends on the density difference between the lesion and the normal breast tissue to observe the overall morphology and characteristics of lesion, but it cannot expose the fine structure and blood supply of the lesion.^[21,22]^ While functional magnetic resonance imaging can reveal the characteristics of tissues from the molecular level, MK represented the average diffusion kurtosis of each ladder direction in space. It was an index to measure the complexity of organizational structure. MK value was proportional to the complexity of the organizational process, and the more complex the structure, the greater the MK value. MD represented the apparent diffusion coefficient value corrected by non-Gaussian distribution.^[23,24]^ ADC value quantified the extent to which molecular dispersion was restricted. Although radiomics and deep learning methods were applied in mammography research,^[25,26]^ MRI still shows more advantages in the diagnosis of breast disease.

For the applications of body DKI, the number and maximum value of b-values varied from each other in previous study. Theoretically, at least 3 b-values were suggested for clinical applications in body DKI. In fact, it may be advantageous to avoid acquiring an excessive number of b-values. The subgroup analysis (number of b-values) in this study showed that the group with 3 - 5 b-values had higher AUC than that of 6 or 7 b-values for MD (*P* = .047). Moreover, the excessive number of b-values might increase the overall scan time, both increasing the likelihood of motion artifact and hindering incorporation of DKI into clinical protocols.

In the DKI studies of brain, it is suggested that the maximum b-value is 2000-2500 s/mm^2^. The use of such a high b-value in other organs outside the brain is limited by the signal-to-noise ratio and faster signal decay of the transverse relaxation. Compared with head coil, the lower sensitivity of flexible surface coil also leads to lower signal-to-noise ratio in tissues.^[27]^ We found that these studies (maximum b-value: 2000–2500 s/mm^2^) had higher AUC than studies with maximum b-value > 2500 s/mm^2^, which was not statistically significant. Therefore, considering the examination time and equipment requirements, it may not be necessary to use an ultra-high b-value of 2500–3000 s/mm^2^.

Although some published papers suggested that DKI was superior to DWI in diagnostic and theraputic evaluation of tumors, Li et al^[28]^ (in ovarian tumors), Roethke et al^[29]^ and Tamura et al^[30]^ (in prostate), Wan Q et al^[31]^ and Das et al^[32]^ (in lung), and Yang L et al^[33]^ (in liver) didn’t report the additional value of DKI superior to DWI. For our results, large-scale prospective studies were needed to further verify our findings in the diagnosis of breast cancer.

## 5. Limitations

Some limitations of this meta-analysis should be noted. First, a small number of studies were included. Our meta-analysis was aimed to assess the role of DKI compared to DWI in breast. Therefore, only studies including both DKI and DWI were evaluated. In addition, studies that didn’t have enough data to construct a 2 *×* 2 contingency table were removed, even if they included the 2 imaging methods mentioned above. Secondly, we compared the diagnostic value of DKI and DWI in the differential diagnosis of breast lesions, but we did not evaluate other important issues, such as tumor grade or prediction of invasion due to the small number of related papers.

## 6. Conclusion

In conclusion, DKI and DWI showed comparable diagnostic performance for the differentiation of breast lesions. On the basis of current evidence, we do not recommend including DKI in routine clinical assessment of breast lesions for the moment. However, considering the advantages and potential of DKI, we still expect further studies with standardized method and optimized imaging protocols to improve the performance of DKI in the diagnosis of breast lesions.

## Author contributions

**Formal analysis:** Yumei Jin.

**Methodology:** Yewu Wang, Jun Zhang.

**Software:** Mou Li, Shaoyu Wang, Huapeng Zhang.

**Supervision:** Bin Song.

**Validation:** Bin Song.

**Writing – original draft:** Yumei Jin.

## References

[1] Torre LA Bray F Siegel RL. Global cancer statistics, 2012. CA Cancer J Clin. 2015;65:87–108. 25651787 10.3322/caac.21262

[2] Kanao S Kataoka M Iima M. Differentiating benign and malignant inflammatory breast lesions: value of T2 weighted and diffusion weighted MR images. Magn Reson Imag. 2018;50:38–44. 10.1016/j.mri.2018.03.012 29545213

[3] Kim S-Y Cho N Hong H. Abbreviated screening MRI for women with a history of breast cancer: comparison with full-protocol breast MRI. Radiology. 2022;305:36–45. 35699580 10.1148/radiol.213310

[4] Singh N Jain S Verma A. Role of diffusion weighted magnetic resonance imaging in prediction of pathological complete response to neoadjuvant chemotherapy in locally advanced breast cancer and its molecular subtypes. Indian J Radiol Imag. 2022;3:332–8. 10.1055/s-0042-1750155 PMC9514905 36177282

[5] Honda M Iima M Kataoka M. Biomarkers predictive of distant disease-free survival derived from diffusion-weighted imaging of breast cancer. Magn Reson Med Sci. 2022;3:1–8. 10.2463/mrms.mp.2022-0060 PMC10552669 35922924

[6] Fang S Zhu J Wang Y. The value of whole-lesion histogram analysis based on field of view optimized and constrained undistorted single shot (FOCUS) DWI for predicting axillary lymph node status in early-stage breast cancer. BMC Med Imag. 2022;22:163. 10.1186/s12880-022-00891-6 PMC9464403 36088299

[7] Thomassin-Naggara I De Bazelaire C Chopier J. Diffusion-weighted MR imaging of the breast: advantages and pitfalls. Eur J Radiol. 2013;82:435–43. 22658868 10.1016/j.ejrad.2012.03.002

[8] Akin Y Uğurlu MU Kaya H. Diagnostic value of diffusion-weighted imaging and apparent diffusion coefficient values in the differentiation of breast lesions, histpathologic subgroups and correlation with prognostic factors using 3.0 Tesla MR. J Breast Health. 2016;12:123–32. 28331748 10.5152/tjbh.2016.2897 PMC5351482

[9] Jensen JH Helpern JA Ramani A. Diffusional kurtosis imaging: the quantification of non-gaussian water diffusion by means of magnetic resonance imaging. Magn Reson Med. 2005;53:1432–40. 15906300 10.1002/mrm.20508

[10] Wu D Li G Zhang J. Characterization of breast tumors using diffusion kurtosis imaging (DKI). PLoS One. 2014;9:e113240. 25406010 10.1371/journal.pone.0113240 PMC4236178

[11] Sun K Chen X Chai W. Breast cancer: diffusion kurtosis MR imaging-diagnostic accuracy and correlation with clinical-pathologic factors. Radiology. 2015;277:46–55. 25938679 10.1148/radiol.15141625

[12] Christou A Ghiatas A Priovolos D. Accuracy of diffusion kurtosis imaging in characterization of breast lesions. Br J Radiol. 2017;90:20160873. 28383279 10.1259/bjr.20160873 PMC5605109

[13] Suo S Cheng F Cao M. Multiparametric diffusion-weighted imaging in breast lesions: association with pathologic diagnosis and prognostic factors. J Magn Reson Imag. 2017;46:740–50. 10.1002/jmri.25612 28139036

[14] Li T Yu T Li L. Use of diffusion kurtosis imaging and quantitative dynamic contrast-enhanced MRI for the differentiation of breast tumors. J Magn Reson Imag. 2018;48:1358–66. 10.1002/jmri.26059 29717790

[15] Huang Y Lin Y Hu W. Diffusion kurtosis at 3.0T as an in vivo imaging marker for breast cancer characterization: correlation with prognostic factors. J Magn Reson Imag. 2019;49:845–56. 10.1002/jmri.26249 30260589

[16] Liu W Wei C Bai J. Histogram analysis of diffusion kurtosis imaging in the differentiation of malignant from benign breast lesions. Eur J Radiol. 2019;117:156–63. 31307642 10.1016/j.ejrad.2019.06.008

[17] Zhou WP Zan XY Hu XY. Characterization of breast lesions using diffusion kurtosis model-based imaging: an initial experience. J X-Ray Sci Technol. 2020;28:157–69. 10.3233/XST-190590 31815728

[18] Li T Hong Y Kong D. Histogram analysis of diffusion kurtosis imaging based on whole-volume images of breast lesions. J Magn Reson Imag. 2020;51:627–34. 10.1002/jmri.26884 31385429

[19] Meng N Wang X Sun J. A comparative study of the value of amide proton transfer-weighted imaging and diffusion kurtosis imaging in the diagnosis and evaluation of breast cancer. Eur Radiol. 2020;31:1707–17. 32888071 10.1007/s00330-020-07169-x

[20] Whiting PF Rutjes AW Westwood ME. QUADAS-2: a revised tool for the quality assessment of diagnostic accuracy studies. Ann Intern Med. 2011;155:529–36. 22007046 10.7326/0003-4819-155-8-201110180-00009

[21] Gentle Corey K Alkhatib H Valente Stephanie A. ASO visual abstract: stage IV non-breast cancer patients and screening mammography: it is time to stop. Ann Surg Oncol. 2022;29:6369. 35849289 10.1245/s10434-022-12132-9

[22] Coffey K Jochelson Maxine S. Contrast-enhanced mammography in breast cancer screening. Eur J Radiol. 2022;156:110513. 36108478 10.1016/j.ejrad.2022.110513 PMC10680079

[23] Park VY Kim Sungheon G Kim E-K. Diffusional kurtosis imaging for differentiation of additional suspicious lesions on preoperative breast MRI of patients with known breast cancer. Magn Reson Imaging. 2019;62:199–208. 31323316 10.1016/j.mri.2019.07.011 PMC6945504

[24] Zhang D Geng X Suo S. The predictive value of DKI in breast cancer: does tumour subtype affect pathological response evaluations? Magn Reson Imag. 2022;85:28–34. 10.1016/j.mri.2021.10.013 34662700

[25] Zhang Y-D Satapathy S Chandra G. Improved breast cancer classification through combining graph convolutional network and convolutional neural network. Inf Process Manag. 2021;58:102439.

[26] Wang S Rao R Venkata C. Abnormal breast detection in mammogram images by feed-forward neural network trained by Jaya algorithm. Fundam Inform. 2017;151:191–211.

[27] Pentang G Lanzman RS Heusch P. Diffusion kurtosis imaging of the human kidney: a feasibility study. Magn Reson Imag. 2014;32:413–20. 10.1016/j.mri.2014.01.006 24582288

[28] Li HM Zhao SH Qiang JW. Diffusion kurtosis imaging for differentiating borderline from malignant epithelial ovarian tumors: a correlation with Ki-67 expression. J Magn Reson Imag. 2017;46:1499–506. 10.1002/jmri.25696 28295854

[29] Roethke MC Kuder TA Kuru TH. Evaluation of diffusion kurtosis imaging versus standard diffusion imaging for detection and grading of peripheral zone prostate cancer. Invest Radiol. 2015;50:483–9. 25867657 10.1097/RLI.0000000000000155

[30] Tamura C Shinmoto H Soga S. Diffusion kurtosis imaging study of prostate cancer: preliminary findings. J Magn Reson Imag. 2014;40:723–9. 10.1002/jmri.24379 24924835

[31] Wan Q Deng YS Lei Q. Differentiating between malignant and benign solid solitary pulmonary lesions: are intravoxel incoherent motion and diffusion kurtosis imaging superior to conventional diffusion-weighted imaging? Eur Radiol. 2019;29:1607–15. 30255258 10.1007/s00330-018-5714-6

[32] Das SK Yang DJ Wang JL. Non-Gaussian diffusion imaging for malignant and benign pulmonary nodule differentiation: a preliminary study. Acta Radiol. 2017;58:19–26. 27055919 10.1177/0284185116639763

[33] Yang L Rao S Wang W. Staging liver fibrosis with DWI: is there an added value for diffusion kurtosis imaging? Eur Radiol. 2018;28:3041–9. 29383522 10.1007/s00330-017-5245-6

